# Differential lung gene expression identified Zscan2 and Bag6 as novel tissue repair players in an experimental COPD model

**DOI:** 10.1371/journal.pone.0309166

**Published:** 2024-08-22

**Authors:** Laura Sánchez Carretero, Àdele Chole Cardeñosa Pérez, Germán Peces-Barba, Sandra Pérez-Rial

**Affiliations:** 1 Respiratory Research Unit, Health Research Institute–Fundación Jimenez Diaz University Hospital, Madrid, Spain; 2 Molecular Genetics Department, Ramón y Cajal University Hospital–IRYCIS, Madrid, Spain; 3 Network Biomedical Research Center for Rare Diseases, Carlos III Health Institute (CIBERER, ISCIII), Madrid, Spain; Concordia University Irvine, UNITED STATES OF AMERICA

## Abstract

Chronic obstructive pulmonary disease is a common chronic lung disease with an ever-increasing incidence. Despite years of drug research and approvals, we are still not able to halt progress or restore normal lung function. Our previous studies have demonstrated that liver growth factor—LGF has an effect on the repair of the affected tissue in a mouse model of cigarette smoke exposure, but by what pathways it achieves this is unknown. The present study aimed to identify differentially expressed genes between emphysematous mice treated with LGF to identify potential therapeutic targets for the treatment of pulmonary emphysema. The emphysema mouse model was induced by prolonged exposure to cigarette smoke. To determine the gene expression profile of the lung in smokers treated or not with LGF, lung messenger RNA gene expression was assessed with the Agilent Array platform. We carried out differentially expressed gene analysis, functional enrichment and validated in treated mouse lung samples. The treated group significantly improved lung function (~35%) and emphysema level (~20%), consistent with our previous published studies. Microarray analysis demonstrated 290 differentially expressed genes in total (2.0-fold over or lower expressed). Injury repair-associated genes and pathways were further enhanced in the lung of LGF treated mice. The expression trends of two genes (Zscan2 and Bag6) were different in emphysematous lungs treated with LGF compared to untreated lungs. Therefore, Zscan2 and Bag6 genes could play a role in regulating inflammation and the immune response in the lung that undergoes partial lung regeneration. However, further studies are necessary to demonstrate this causal relationship.

## Introduction

Chronic obstructive pulmonary disease (COPD) affects more than 380 million people around the world and is currently the third most common cause of mortality [1]. COPD is a progressive lung disease characterized by persistent airflow limitation in chronic bronchitis and tissue damage with substantial reduction in solid lung texture and airspace enlargement in emphysema, eventually leading to irreversible airflow limitation and persistent respiratory symptoms [2]. While the exact mechanisms underlying the pathophysiology are not known, it is generally accepted that COPD is subject to genetic and environmental control. Cigarette smoke exposure (CSE) remains the greatest modifiable risk factor in the Western world [3].

The current treatment for COPD involves the administration of inhaled bronchodilators and corticosteroids. However, there is no treatment capable of stopping the disease from progressing or repairing the lung [4]. Cell‒cell interactions between alveolar epithelial cells and other cell types are promoted through the release of growth factors, including liver, keratinocyte, epithelial or vascular endothelial growth factor [5, 6]. Repair mechanisms to restore normal airway architecture are inefficient in COPD patients. Consequently, the use of growth factors as promoters of cellular proliferation and differentiation mechanisms appeared to be a promising strategy for stimulating tissue repair under injury conditions [7]. Identifying new players involved in tissue repair could provide valuable information on possible therapeutic targets for COPD.

Liver growth factor (LGF) is an albumin-bilirubin complex of 64 kDa with mitogenic properties that was purified in 1986 in rat liver by the group of Dr. Díaz-Gil [8]. The antifibrotic and antioxidant properties of LGF, as well as regenerative effects, have been described in various rodent models [9, 10]. Furthermore, we have already described the beneficial effects of LGF in a rat model by cadmium chloride (ClCd_2_)-induced pulmonary fibrosis [11] and more recently in AKR/J [12] or C57BL/6J [13, 14] mice with CSE-induced emphysema. However, the genes that play a role in this effect have not been previously described.

Recent advances in genomics have enabled genome-wide messenger RNA (mRNA) profiling to provide new insights into the pathogenesis of COPD [15–21]. While these studies identify genes associated with disease phenotypes or smoking, additional in vivo studies are needed to clarify the mechanism and determine causality. Analysis of microarray data demonstrated a large number of small airway epithelial genes that were significantly over or lower expressed in response to smoking. While by no means complete, this subset of genes modulated by smoking provides a working list of potential targets for therapeutic intervention to prevent the development of COPD and to evaluate the efficacy of COPD-related therapies.

The availability of whole-genome transcriptomic signatures in lung tissue allows comparisons between treated and untreated emphysematous mice. The objectives of this study were to compare and contrast the molecular changes in mouse models after LGF treatment with the molecular changes in the lungs caused by cigarette smoke.

## Material and methods

### Chronic cigarette smoke exposure mouse model

This study was carried out in strict accordance with the recommendations in the Guide for the Care and Use of Laboratory Animals of the National Institutes of Health. The Committee on the Ethics of Animal Experiments of the Health Research Institute- Fundación Jiménez Díaz University Hospital approved the protocol (Protocol Number: 322/14). All surgery was performed under ketamine/xylazine (1/3) anesthesia, and all efforts were made to minimize suffering. The study was reported in accordance with ARRIVE guidelines (https://arriveguidelines.org).

**Fig 1:**
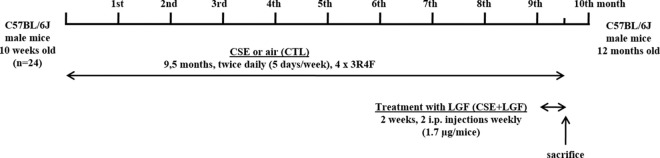
Scheme methodological procedure. Representative scheme showing the methodological procedure of chronic cigarette smoke exposure (CSE) followed by the intraperitoneal administration of liver growth factor (LGF) once the lung was already damaged to C57BL/6J male mice.

LGF treatment of the chronic CSE-induced emphysema model was assessed as previously described [22]. Twenty-four adult inbred males, 10-week-old C57BL/6J mice (Charles River Laboratories, France) susceptible to smoking-induced emphysema were used. Mice were divided into air-exposed (control CTL, n = 8) and cigarette smoke-exposed (CSE, n = 16) groups, of which half were treated with the growth factor LGF (CSE+LGF, n = 8) two weeks before the sacrifice of the animals once the lung was already damaged (Fig 1). In this study, the group of control mice treated with LGF has not been included, since in previous studies we have verified that LGF has no effect on the measured parameters [13].

Smoking animals were exposed to a mainstream CSE of 4 unfiltered cigarettes (3R4F, University of Kentucky, Lexington, KY) per day (15 minutes per cigarette with 5 minutes smoke free intervals between them), twice daily (5 days/week) over a period of 9.5 months. s. Mainstream CSE was generated by an exposure system of own manufacture and was drawn into the chamber using a peristaltic pump following the previously published methodology [23] that reaching concentrations of 250 mg TPM/m3 (Dust Track Model 8520, TSI Inc.). Non-smoking mice were exposed to room air. The efficiency of smoking exposure was evaluated spectrophotometrically by randomly measuring the blood levels of carboxyhemoglobin (COHb) in the animals with an IL 682 CO-Oximeter (Instrumentation Laboratory) following previously described methodology [23]. The mean percent COHb of control mice was 0.2 ± 0.05%, and that of mice exposed to smoke chamber was within the nontoxic 13.9 ± 0.02% value, confirming the correct exposure to tobacco smoke. Nine months after CSE, when emphysema was once established, half of the smoker group animals were treated with four intraperitoneal injections (twice a week for 2 wk.) of 150 μl of a solution containing 1.7 μg LGF per mouse (CSE+LGF group) or saline solution (CSE group). This optimal dose of LGF has been used in different model systems using an identical or similar schedule [14].

Lung function was studied by measuring changes in quasistatic lung compliance (C_L_, ml/cmH_2_O) and maximum expiratory volume (V_max_, ml) at a pressure of 25 mbar after inspiratory pressure-controlled ventilation. Mice were tracheotomized and connected to the ventilator according to the previously published methodology [23]. After 9.5 months, mice were sacrificed by an intraperitoneal injection with an overdose of pentobarbital. Lung tissue was obtained from the cardiopulmonary block after tying the right bronchus. Thus, the right lung lobes were frozen, and the left lung lobe was fixed by filling it with 4% buffered formalin solution at a constant airway pressure of 25 cmH_2_O for 24 h. Fixed lungs were kept in formalin solution until they were paraffin embedded, cut into 5-μm-thick sections using a rotary microtome HM325 (Microm International GmbH). Morphometric analysis was made following the previously published methodology [23] to confirm emphysema by measuring the mean linear intercept (L_M_, μm) and the alveolar internal area (AIA, μm^2^) in the alveolus.

### RNA extraction and RNA quality control of lung tissue samples

Total RNA of the right lung lobe tissue from 6 mice per experimental group was extracted using TRIzol (Invitrogen, Carlsbad, CA, USA) and an RNeasy^®^ Plus Mini Kit (Qiagen GmbH, Germany), yielding 6 μg from each tissue. RNA quantity and purity tests from each sample were measured by a NanoDrop spectrophotometer (ND-1000), including an OD_260/280_ ratio of 1.7 to 2.3. RNA integrity and genome DNA contamination tests were assessed by standard denaturing formaldehyde gel electrophoresis and Agilent 2100 Bioanalyzer (measure RNA integrity number). The quality of the RNA labelling was verified by hybridization to a test chip, and only test chips with a 3′ to 5′ ratio of < 3 were deemed satisfactory. RNA from each cell sample was sent to Arraystar Inc. (Rockville, MD, USA) for microarray analysis.

### Lung gene expression microarrays

To determine the gene expression profile of the lung in smokers, Arraystar, Inc. (Rockville, MD, USA) assessed RNA for gene expression with the Agilent Array platform (Agilent Technologies, Inc., Santa Clara, CA, USA). Sample preparation and microarray hybridization were performed based on the Agilent One-Color Microarray-Based Gene Expression Analysis protocol. Briefly, total RNA from each sample was linearly amplified, transcribed into fluorescent complementary RNA (cRNA) and labelled with Cy3-UTP using the manufacturer’s Agilent’s Quick Amp Labelling protocol (version 5.7). The labelled cRNAs were purified using an RNeasy Mini Kit (Qiagen GmbH, Germany). The concentration and specific activity of the labelled cRNAs (pmol Cy3/μg cRNA) were measured by a NanoDrop ND-1000 spectrophotometer. One microgram of each labelled cRNA was fragmented by adding 11 μl of 10-× blocking agent plus 2.2 μl of 25-× fragmentation buffer; and then heated at 60°C for 30 min. Finally, 55 μl of 2-× GE hybridization buffer was added to dilute the labelled cRNA. The labelled cRNAs were hybridized onto the Whole Mouse Genome Oligo Microarray (4 x 44K) in Agilent´s SureHyb hybridization chambers, printed using Agilent´s 60-mer SurePrint technology. The array includes probes representing ∼39,000 full-length mouse genes and transcripts, all with public domain annotations. One hundred microliters of hybridization solution was dispensed into the gasket slide and assembled into the gene expression microarray slide. The slides were incubated for 17 hours at 65°C in a hybridization oven. After washing, slides were scanned with the Microarray Scanner G2505C.

### Microarray data analysis

Image processing and data extraction and analysis also were performed by Arraystar Inc. (Rockville, MD, USA) using their established protocols. Agilent Feature Extraction software (v11.0.1.1) was used to analyze acquired array images. Quantile normalization and subsequent data processing were performed using Agilent GeneSpring GX (v12.1) software as follows: (1) per array, by dividing the raw data by the 50th percentile of all measurements; and (2) per gene, by dividing the raw data by the median of the expression level for the gene in all samples. The distribution of log2 ratios among all samples was nearly the same. After quantile normalization of the raw data, genes in at least 12 out of 18 samples were chosen for differentially expressed gene (DEG) screening. Further data analysis was also performed using Agilent GeneSpring GX software, and hierarchical clustering analysis was performed to show the distinguishable gene expression profiling among samples. DEGs with statistical significance between two groups were identified through volcano plot filtering. The threshold was fold change (FC) ≥ 2.0 and p value ≤ 0.05. The p values were further adjusted using the false discovery rate (FDR) approach, according to the Benjamini ‑ Hochberg (BH) method.

### Functional enrichment of DEGs and selection of hub genes

GO functional analysis (Gene Ontology Biological Processes, www.geneontology.org) and pathway analysis were applied to determine the role of these DEGs in these GO terms or biological pathways. Based on the latest KEGG (Kyoto Encyclopedia of Genes and Genomes, www.genome.jp/kegg) database, pathway enrichment analysis of the DEGs was performed. To identify the pathways associated with the groups, tools in DAVID (Database for Annotation, Visualization and Integrated Discovery, https://david.ncifcrf.gov) were used to screen the pathways enriched in the DEGs from the samples using the Expression Analysis Systematic Explorer score (a modified Fisher’s exact t ‑test) with BH multiple testing correction. A KEGG pathway with a BH‑ corrected p <0.05 was considered to be significantly enriched. The DAVID tools were used to identify enriched functional-related hub genes.

### Quantitative real-time PCR for validation of the hub genes

The results of the 10 hub genes selected among those that demonstrate a high discriminatory value will be confirmed by the quantitative technique of polymerase chain reaction with reverse transcriptase (qRT‒PCR) following the standard protocol described in previous articles from our group [13] and using the same RNA samples that had been used for the microarray analysis. First strand cDNA was synthesized from 2 μg of RNA in a 100 μl reaction volume using the TaqMan Reverse Transcriptase Reaction Kit (Applied Biosystems) with random hexamers as primers. The cDNA was diluted 1:100 or 1:50, and each dilution was run in triplicate wells. Five microliters was used for each TaqMan PCR in a 25 μl final reaction volume using premade kits from Applied Biosystems. The relative quantity (ΔΔCt) was determined using the algorithm provided by Applied Biosystems using the beta-actin (Actb) gene as the internal control to normalize the expression of the target genes mentioned above and the average value for control non-smokers as the calibrator. The Actb probe was labelled with VIC, and the probes for the genes of interest were labelled with FAM. The PCRs were run in an Applied Biosystems Sequence Detection System 7500 by 40 cycles of 15 seconds at 94°C, 30 seconds at 55°C, and 30 seconds at 72°C. For comparison purposes, the data for each individual were normalized to the median across all control non-smokers and smoker samples, as was done with microarray data (see above).

### Statistical analysis

Data are presented as the mean ± standard deviation (SD). Normally distributed data were assessed for significance by Student’s t test or ANOVA as appropriate. Data that were not normally distributed were assessed for significance using the Mann–Whitney U test or the Kruskal–Wallis test with Dunn’s post-test for multiple comparisons as appropriate. Prism version 8 (GraphPad) was used for data analysis. A two-sided p value less than 0.05 was considered statistically significant.

## Results

### LGF reverts emphysema in the CSE model: Functional and morphometric studies

**Fig 2:**
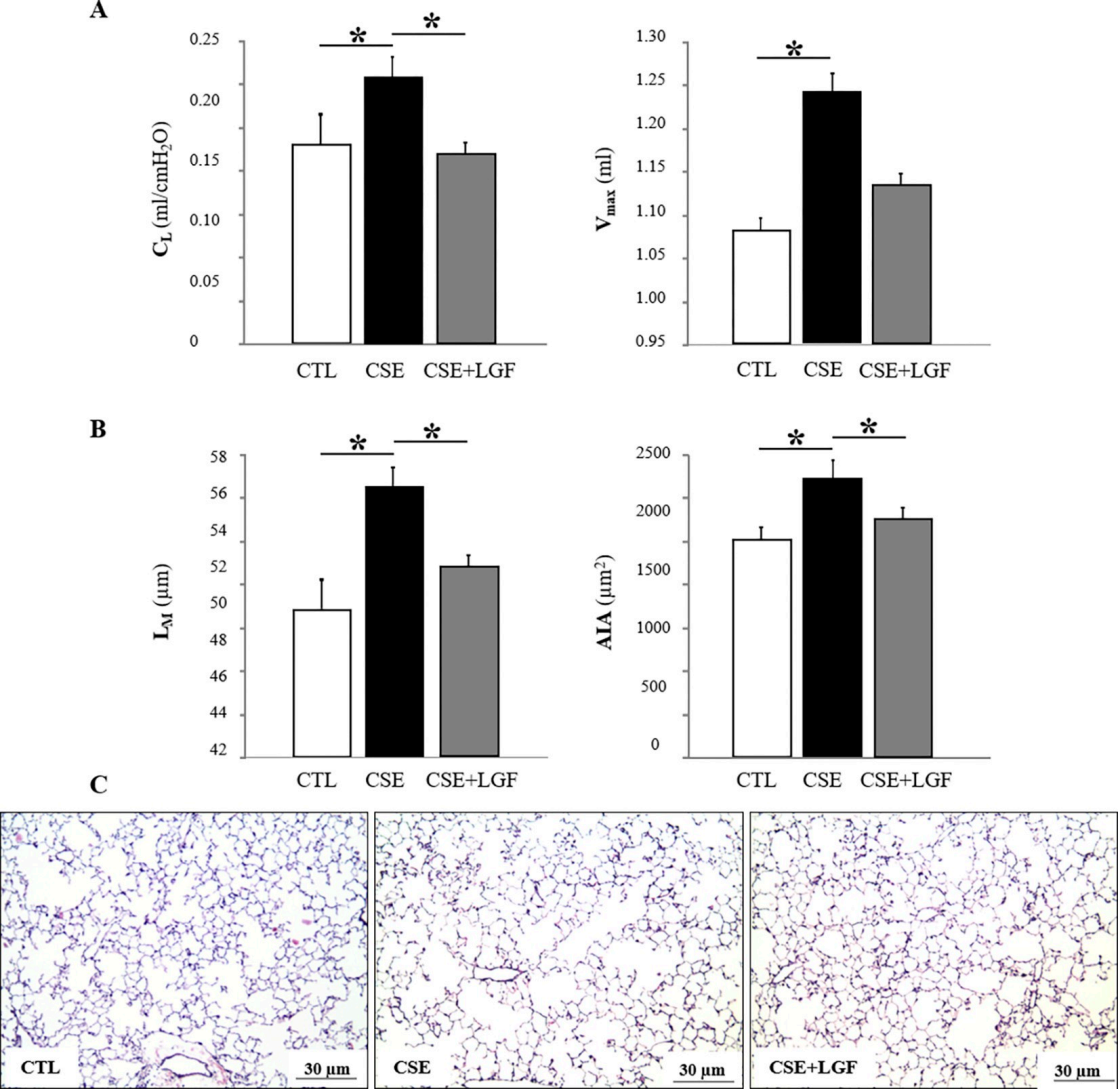
LGF revert emphysema in a CSE model. **(A)** Changes in lung compliance (C_L_, ml/cmH2O) and maximum expiratory volume (V_max_, ml) in the lungs of air-exposed group (CTL, white bar) compared to CSE mice (black bar) and CSE+LGF mice (grey bar). **(B)** Mean values of the mean linear intercept (L_M_, μm) and alveolar internal area (AIA, μm2) in the lungs of the air-exposed group (CTL, white bar) compared to cigarette smoke-exposed mice (CSE, black bar) and cigarette smoke-exposed LGF treated mice (CSE+LGF, grey bar). **(C)** Representative images (x10 magnification) corresponding to lung samples (30 μm calibration bar). Data are expressed as mean ± SD, *p≤005.

Functionally, pulmonary emphysema induced by chronic CSE was reversed by LGF treatment, as measured by lung compliance (C_L_, ml/cmH_2_O) and maximum expiratory volume (V_max_, ml) at 25 mbar (Fig 2A). Mean ± SD, C_L_ = 0.158 ± 0.023 ml/cmH_2_O in the CTL group *vs*. 0.23 ± 0.012 ml/cmH_2_O in the CSE group. p = 0.029 *vs*. 0.15 ± 0.003 ml/cmH_2_O in the CSE+LGF group, p = 0.010; V_max_ = 1.084 ± 0.01 ml in the CTL group *vs*. 1.345 ± 0.02 ml in the CSE group, p = 0.005 *vs*. 1.140 ± 0.015 ml in the CSE+LGF group. Note that loss of lung function is significantly reverted in the emphysematous mice after treatment with LGF in terms of C_L_, not significantly in the V_max_.

Morphometrically, as measured by the mean linear intercept (L_M_, μm) and alveolar internal area (AIA, μm^2^). LGF was able to revert emphysema in CSE+LGF mice compared to CSE mice, which presented enlargement of the alveolar space. CTL mice presented normal alveolar architecture. Mean ± SD, L_M_ = 48.2 ± 0.54 μm in the CTL group *vs*. 55.6 ± 0.32 μm in the CSE group, p = 0.016 *vs*. 52.2 ± 0.58 μm in the CSE+LGF group, p = 0.013. AIA = 1745.2 ± 22.3 μm^2^ in the CTL group *vs*. 2279.8 ± 28.4 μm^2^ in the CSE group, p = 0.017 *vs*. 1920.6 ± 15.6 μm^2^ in the CSE+LGF group, p = 0.016 (Fig 2B). Note that the alveolar space is significantly reverted in the emphysematous mice treated with LGF. Histological sections of lungs in CTL, CSE and CSE+LGF mice are shown in Fig 2C. Note that the size of the alveoli is reverted in CSE+LGF compared to CSE (middle) and CTL (left) mice.

### Lung tissue transcriptome signature of LGF treatment

**Fig 3:**
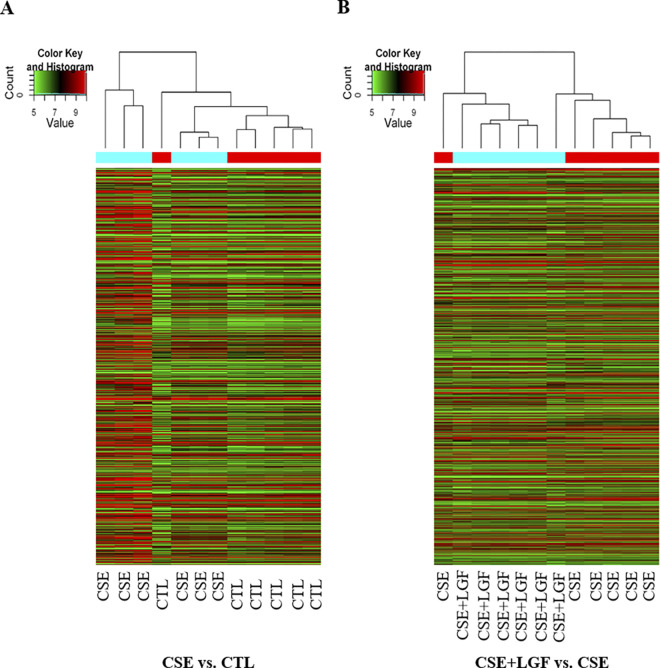
Heat map and supervised hierarchical clustering for DEGs. The dendrograms show the relationships between the expression levels of CSE group vs. CTL group samples (A) and CSE+LGF group vs. CSE samples (B). “Red” indicates high relative expression, and “green” indicates low relative expression. Cluster analysis arranges samples into groups based on their expression levels.

To identify the possible therapeutic target of COPD treatment, we utilized transcriptome sequencing to measure the gene expression variation in control, emphysema and LGF-treated emphysema. A **heat map and supervised hierarchical clustering analysis** were performed on DEGs (Pass Volcano Plot) representing mRNA expression profiles of most changed genes (Fig 3). Cluster analysis arranges samples into groups based on their expression levels, which allows us to hypothesize the relationships among samples. The dendrogram shows the relationships among the expression levels of samples. “Red” indicates high relative expression, and “green” indicates low relative expression. Some of the clusters appear with interspersed patterns. This may be due to the biological heterogeneity of lung tissue samples, which may contain different cell subtypes, each with its own distinct gene expression profile. Moreover, even those cells can be in different physiological (such as proliferation, differentiation) or pathological states, which can lead to interspersed differential gene expression. In addition, variability in response to treatment may cause interspersed patterns in gene expression. Another explanation could be that these represented genes participate in multiple signaling pathways or biological processes that can be co-regulated in complex ways, leading to the appearance of interspersed clusters when these pathways are differentially activated.

**Fig 4:**
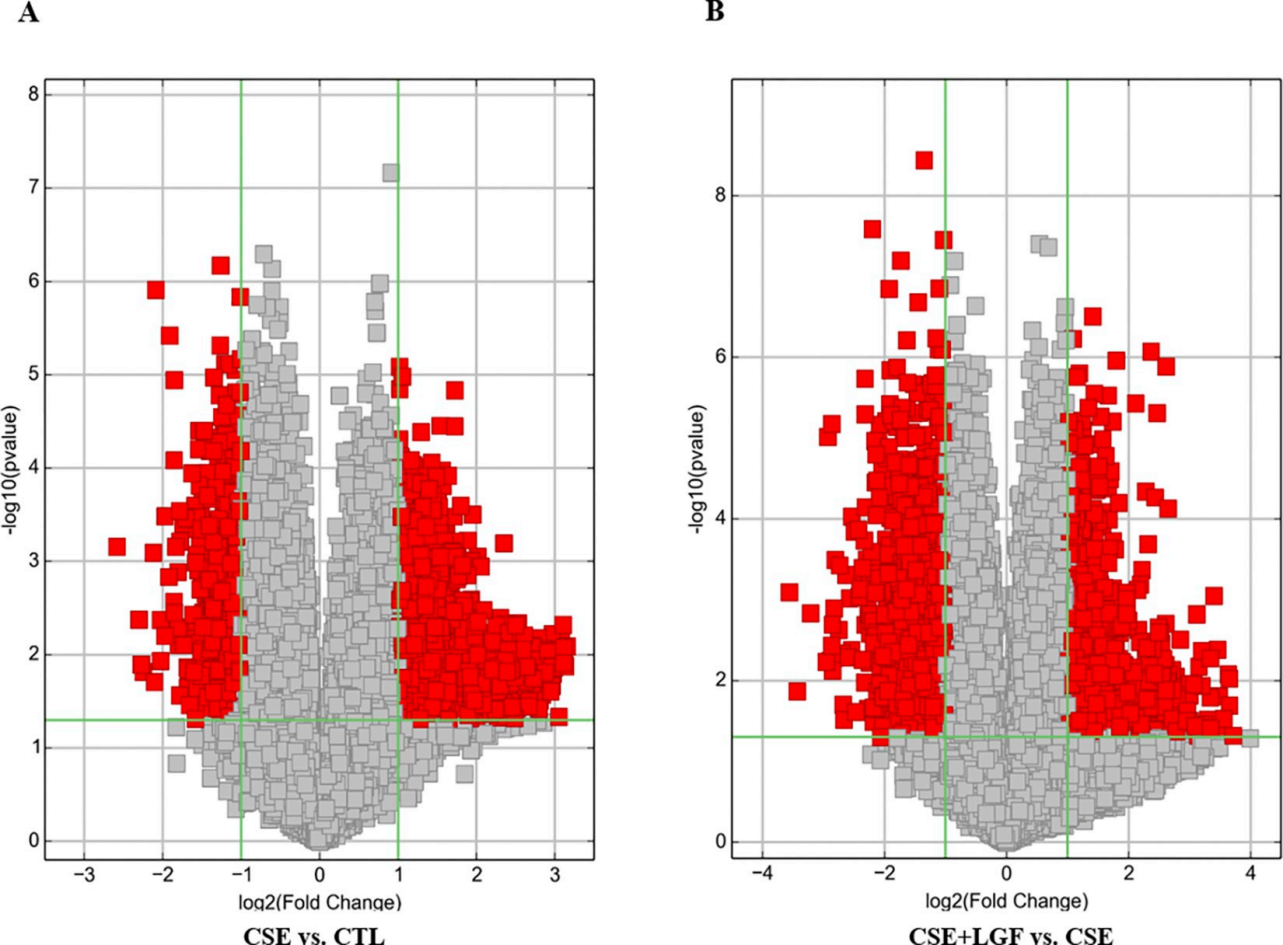
Volcano plot filtering on DEGs. They are constructed using Fold Change (FC) ≥ 2.0 (or log2 (2) = 1) and p-value ≤ 0.05 (-log10 (0.05) ≈ 1.3) and thus allow you to visualize the relationship between magnitude of change and statistical significance. The red point in the plot represents the differentially expressed genes (right points over expressed and left points lower expressed) with statistical significance between CSE vs. CTL group (A) and CSE+LGF vs. CSE group (B).

**Volcano Plot filtering** is useful tool for visualizing the differential expression between two different conditions (magnitude of change (Fold Change -FC) and variability (p-value, statistical significance)). They allow the isolation of subsets of genes based on these values (Fig 4). The vertical lines correspond to 2.0-fold over and lower expressed genes, and the horizontal line represents a p-value of 0.05. Therefore, the red dot in the plot represents the differentially expressed genes with statistical significance.

**Fig 5:**
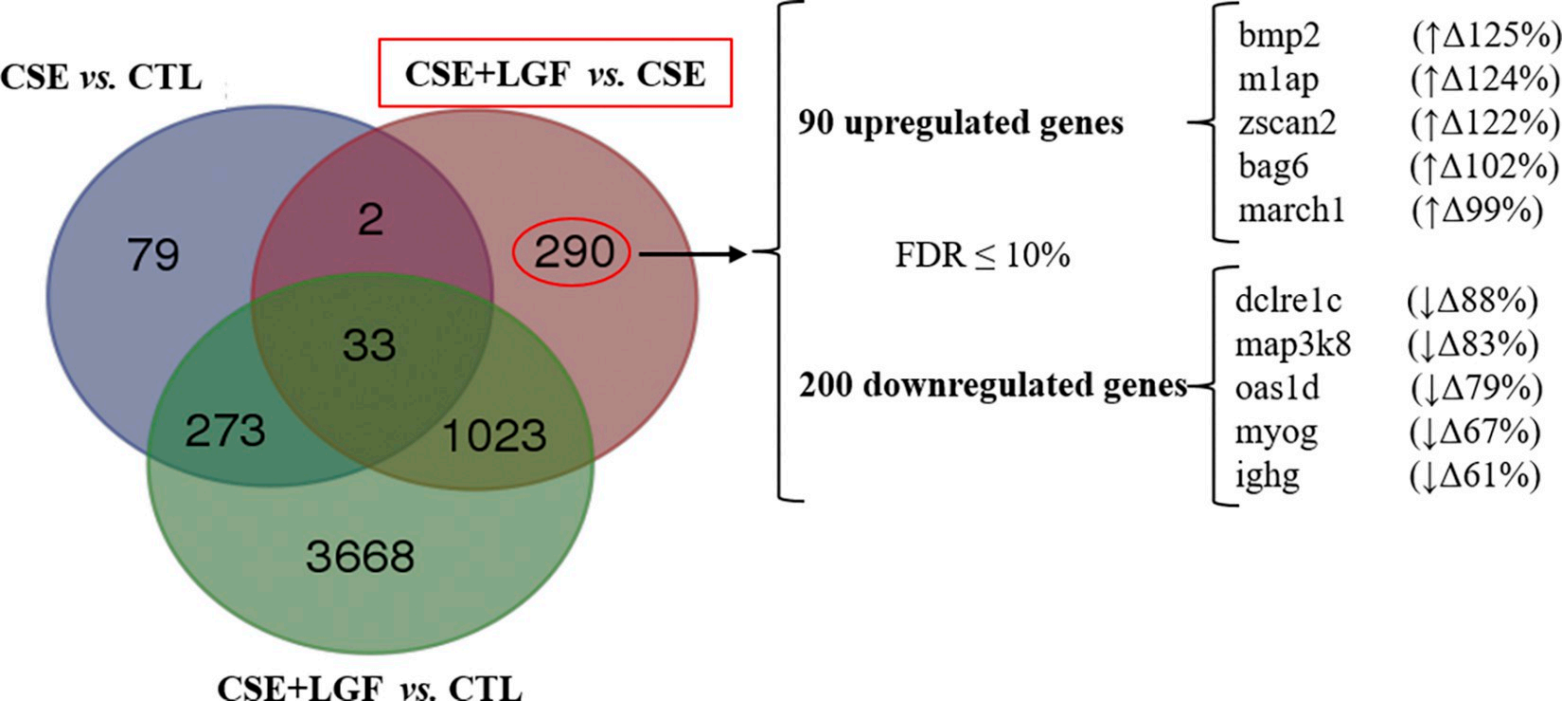
Venn diagram. Overlap of LGF treatment signature with murine smoking signatures. Venn diagram showing the genes differentially expressed in tobacco (CSE) vs. control (CTL); CSE+LGF vs. CSE and CSE+LGF vs. CTL. The diagram revealed 290 mRNAs characteristic of the LGF treatment expression.

**Venn diagram** showing the overlap of the LGF treatment signature with murine smoking genes differentially expressed in smokers (CSE) *vs*. non-smokers (CTL); CSE+LGF *vs*. CSE and CSE+LGF *vs*. CTL (Fig 5). As can be seen in Fig 5, three different comparative analyzes were performed, identifying the differentially expressed mRNAs for each subgroup.

Gene expression analysis of the lung tissue revealed that emphysematous mice treated with LGF showed significant over expression of 90 genes as well as a lower expression of 200 more genes (where the most significant, FDR <10%). From the total 290 genes, we select the 10 DEGs with the highest FC (Table 1).

**Table 1:** Top 10 DEGs for LGF treatment signature.

Gene	ID	Description	Expression	Fold Change	p-value	FDR
**m1ap**	110958	meiosis 1 associated protein	over	3.164	4.19E-05	3.42E-03
**zscan2**	22691	zinc finger and SCAN domain containing 2	over	2.230	1.74E-06	1.08E-03
**bmp2**	12156	bone morphogenetic protein 2	over	2.217	9.22E-06	1.32E-02
**march1**	72925	membrane-associated ring finger 1	over	2.124	7.87E-04	1.22E-02
**bag6**	224727	bcl2-associated athanogene 6	over	2.114	5.61E-05	3.69E-03
**ighg**	380794	immunoglobulin heavy chain	lower	3.948	3.90E-03	2.79E-02
**oas1d**	100535	2´-5´oligoadenylate synthetase 1D	lower	3.192	2.13E-04	6.71E-03
**myog**	17928	myogenin	lower	2.558	7.32E-03	3.95E-02
**dclre1c**	227525	DNA cross-link repair 1C	lower	2.527	5.52E-04	1.04E-02
**map3k8**	26410	mitogen-activated protein 3 kinase 8	lower	2.368	1.78E-02	1.82E-02

Representation of top 10 genes potentially involved in the reversal of the emphysema observed in CSE mice treated with LGF. Genes are classified by FC, p value and FDR.

**Fig 6:**
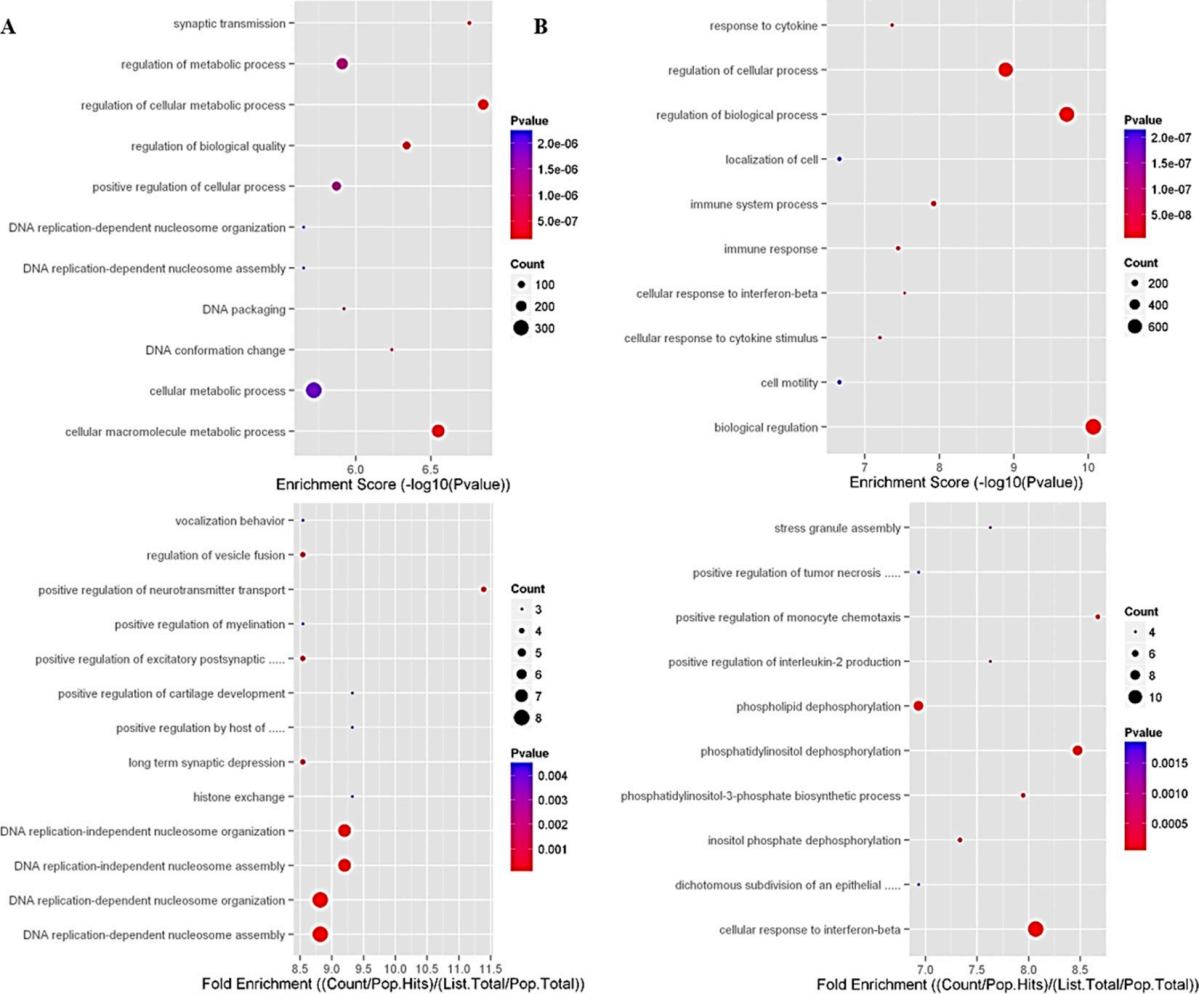
Gene ontology (GO) -based analysis of DEGs–Molecular function categorization between CSE+LGF vs. CSE group. Effect of LGF on gene expression in the treatment of emphysema. Genes differentially expressed between CSE+LGF and CSE mouse lungs are represented by their molecular functional categorization: (A) over expressed, (B) lower expressed.

**Fig 7:**
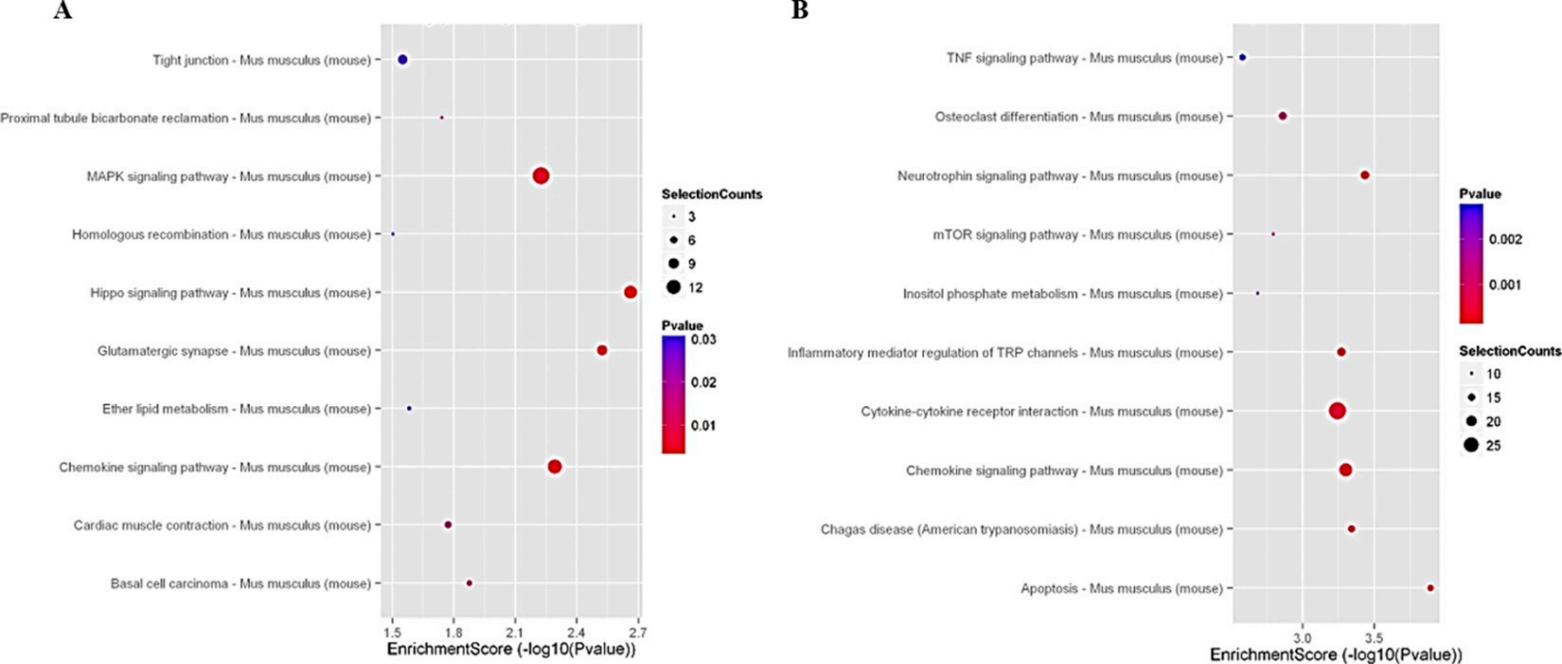
Pathway analysis of DEGs. Significative pathways of genes over expressed (A) or lower expressed (B) between CSE+LGF and CSE groups.

**Functional analysis** associated with the DEGs with the different most significant categories of **Gene Ontology (GO)** for over and lower expressed genes (Fig 6) of the CSE+LGF group *vs*. the CSE group. **Significant pathways of DEGs** over and lower expressed (Fig 7) between the CSE+LGF and CSE groups.

Gene ontology processes and gene sets enriched among treated or untreated murine smoking overlapping gene-associated mRNAs (Table 2).

**Table 2:** Gene ontology processes.

Gene ontology (GO) pathway	Fold Enrich.	p-value	FDR	Total genes
GO:0009790~embryodevelopment	4.64E+16	0.055	5.89E+15	BMP2, EHMTI, BAG6, OAS1D
GO:0045087~innate immune response	2.28E+16	0.032	4.03E+16	IGHG, MEFV, IFITM1, FCNB, OAS3, DEFB45, SLPI, CLEC4D, PADI4, TBKBP1
GO:0006468~protein phosphorylation	2.07E+16	0.024	3.17E+16	ALPK1, BMP2, CAMK1G, TAOK3, TRIB3, PDIK1L, MAST1, GM6729, MAP3K8, LMTK3, ROR2, DYRK2, LRRK2
GO:0031497~chromatin assembly	2.03E+16	0.094	7.88+15	MIAP, RBBP4
GO:0048312~intracellular distribution of mitochondria	2.03E+16	0.094	7.88E+15	SYNJ2, LRRK2
GO:0007155~cell adhesion	1.89E+16	0.083	7.47E+15	RNASE10, COL19A1, CD33, ADAM2, MYBPH, COL28A1, VCAN, VMP1, CD6, SELPLG
GO:0030154~cell differentiation	1.64E+16	0.084	7.48E+15	M1AP, BMP2, ZSCAN2, SETX, SUV39H2, BAG6, COL19A1, MARFI, ROR2, MYOG, CATSPERG2, BMP7, LRRK2, CHD5
GO:0010951~negative regulation of endopeptidase activity	1.37E+16	0.020	2.69E+15	SLPI, STFA1, STFA2
GO:0045071~negative regulation of viral genome replication	8.58E+15	0.047	5.33E+16	IFITM1, OAS3, SLPI
GO:0043967~histone H4 acetylation	8.58E+15	0.047	5.33E+16	MYOG, CHD5, EP400
GO:0034504~protein localization to nucleus	7.85E+15	0.055	5.93E+15	CNEP1R1, BMP7, IRS1
GO:0000723~telomere maintenance	7.23E+15	0.064	6.48E+15	DCLRE1C, POT1A, TERC
GO:0051276~chromosomeorganization	7.23E+15	0.064	6.48E+15	DCLRE1C, SMCHD1, POT1A
GO:0006481~C-terminal protein methylation	6.10E+15	0.032	4.04E+15	LCMT2, ICMT
GO:0030509~BMP signaling pathway	4.21E+15	0.069	6.77E+15	MEGF8, BMP2, ROR2, BMP7
GO:0001503~ossification	3.78E+15	0.089	7.70E+15	BMP2, IFITM1, MYOG, BMP7
GO:0016568~chromatin modification	3.74E+15	0.091	7.79E+15	EHMT1, BAG6, MTF2, PADI4
GO:0016569~covalentchromatin modification	3.12E+15	0.008	1.25E+16	EHMT1, RBBP4, BAG6, MTF2, MSL1, PADI4, CHD5, EP400, SUV39H2
GO:0046578~regulation of Ras protein signal transduction	3.05E+15	0.063	6.45E+15	PLCE1, ICMT
GO:0090527~actin filament reorganization	3.05E+15	0.063	6.45E+15	FRYL, WHAMM
GO:0002376~immune system process	2.88E+15	0.003	4.76E+15	DCLRE1C, MARCH1, BAG6, MEFV, IFITM1, FCNB, MAP3K8, OAS3, SLPI, CLEC4D, PADI4, TBKBP1
GO:0042742~defense responseto bacterium	2.82E+15	0.062	6.38E+15	IGHG, HAMP2, DEFB45, SLPI, CLEC4D, PLAC8
GO:0060485~mesenchyme development	2.29E+15	0.084	7.48+15	BMP2, BMP7
GO:0045109~intermediate filament organization	1.31E+15	0.022	2.91+16	KRT2, DSP, NEFL
GO:0006302~double-strand break repair	5.81E+14	0.031	3.93E+16	DCLRE1C, PARP9, MARF1, SETX
GO:0048839~inner ear development	5.39E+14	0.038	4.56E+16	ATP2B2, BMP2, HPCA, LIN7A
GO:0010466~negative regulation of peptidase activity	3.85E+13	0.041	4.83E+15	NGP, SLPI, COL28A1, WFDC18, STFA2
GO:0006955~immune response	2.58E+03	0.024	3.18+15	MARCH1, ENDOU, CD274, CXCL9, OAS3, CCL27A, SLPI, OTUD7A, OAS1D

Gene ontology processes (GO) and gene sets enriched among treated or untreated murine smoking overlapping gene-associated mRNAs

### qRT‒PCR to verify the RNA-seq results

**Fig 8:**
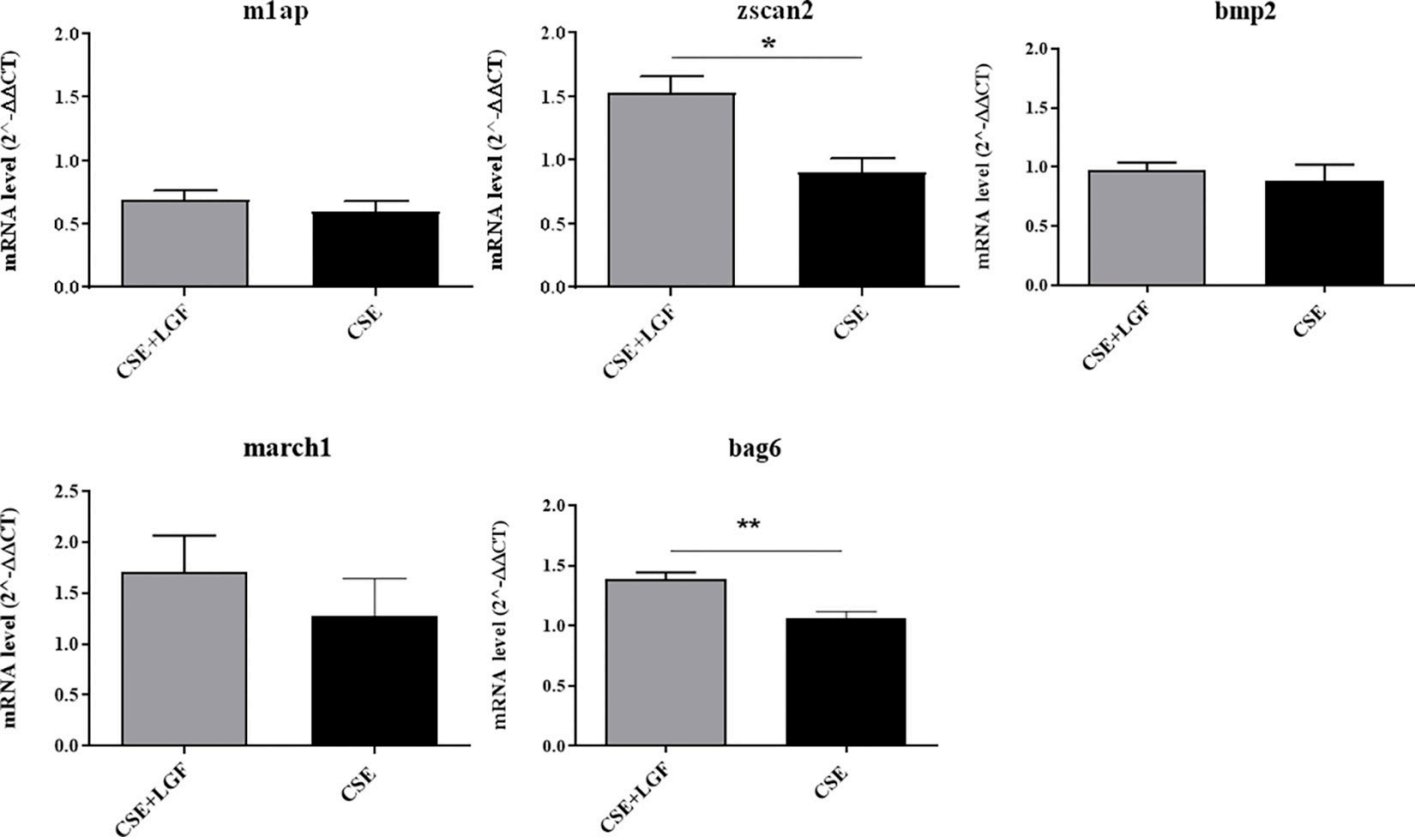
Over expressed genes. qRT-PCR for gene expression of zscan2 and bag6 were sharply increased in CSE+LGF group compared to CSE group. The LGF treatment revealed over expression the mRNA of these specific markers. Data are expressed as mean ± SD, *p≤005, **p≤001.

**Fig 9:**
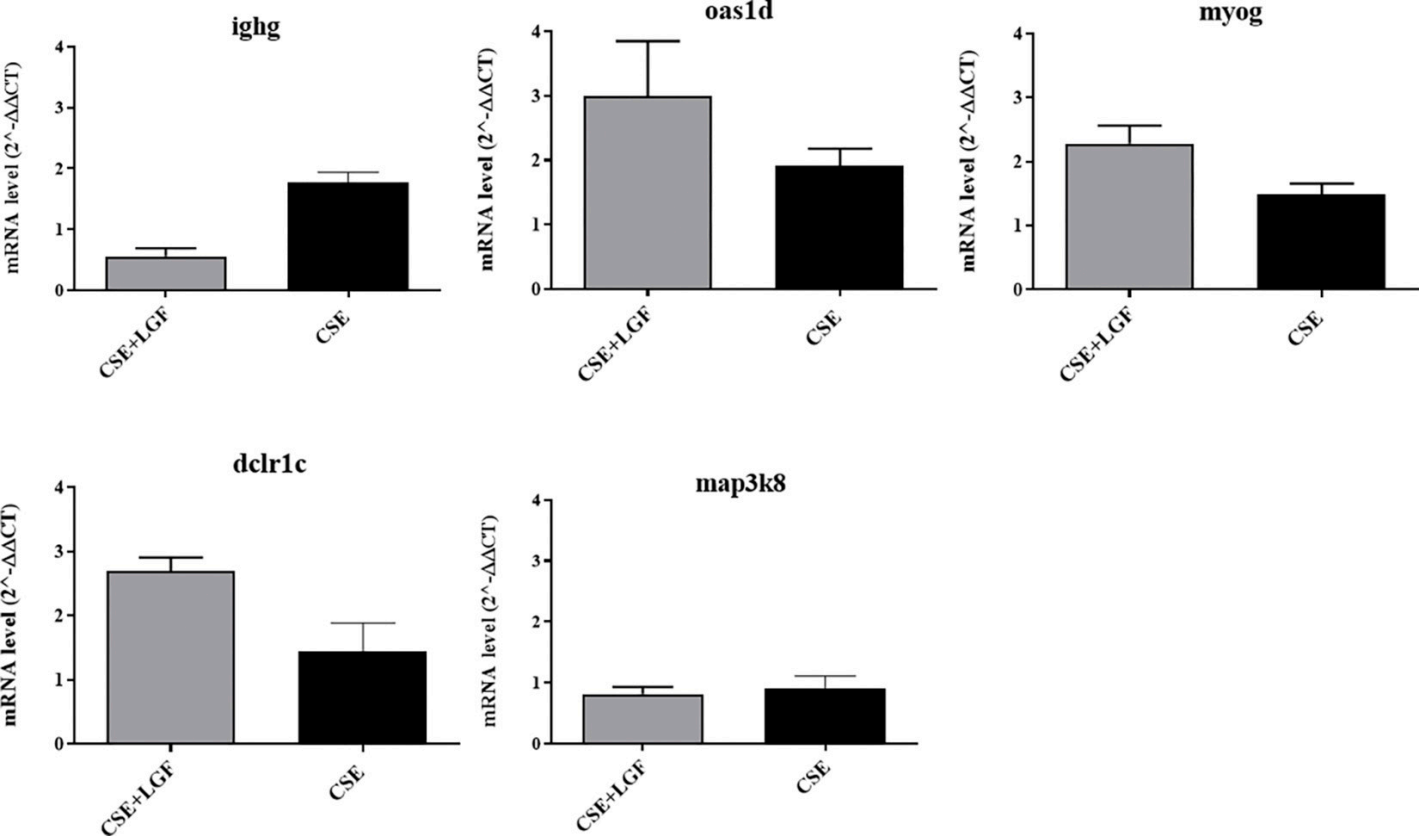
Lower expressed genes. qRT-PCR for gene expression of lower expressed genes between CSE+LGF group and CSE group. There are no significant differences between groups in the genes studied.

Instructed by the RNA-seq results, we further studied the 10 most significantly changed genes in different groups of study. qRT‒PCR was applied to investigate the immune response of different groups. Changes in mRNA levels were measured for the following genes: m1ap, Zscan2, bmp2, march1, and Bag6 (over expressed in the LGF-treated group *vs*. the CSE group, Fig 8) and ighg, oas1d, myog, dclre1c and map3k8 (lower expressed, Fig 9). The gene expression of Zscan2 and Bag6 was sharply increased in the CSE+LGF group compared to the CSE group. Representing mean ± SD, for Zscan2, we found values of 1.524 ± 0.323 in the CSE+LGF group *vs*. 0.903 ± 0.242 in the CSE group, p = 0.0303. For Bag6, 1.390 ± 0.119 in the CSE+LGF group *vs*. 1.061 ± 0.138 in the CSE group, p = 0.0087 (Fig 9). LGF treatment over expressed the mRNA of these specific markers.

## Discussion

COPD is a heterogeneous disease in which chronic bronchiolitis and emphysema are the most important phenotypes and continue to be the leading causes of mortality worldwide [24]. Bronchodilators and anti-inflammatory agents provide the main treatment for patients with COPD; however, they have poor treatment results. A better understanding of the pathophysiology of disease development is recognized as increasingly clinically important, and the identification of critical therapeutic targets specifically for COPD is critically important. Liver growth factor (LGF) has demonstrated utility in an experimental emphysema model that is capable of reversing functional and morphometric changes induced by tobacco smoke [12, 13]. What remains unclear is the mechanism through which it exerts its therapeutic effect on the lung.

With the development of sequencing technology and bioinformatic analysis, the mRNA network hypothesis may partially illustrate disease onset and progression. Despite an increasing number of studies on mRNA networks, the molecular mechanisms of COPD are not yet fully understood [25]. RNA therapeutics offer high specificity and represent safer and reversible alternatives to DNA-based gene therapies. RNA drugs may offer unique opportunities to expand the spectrum of therapeutic targets, several of which are approved or currently in clinical trials. In the present study, we utilized transcriptome array analysis of lung tissue from three groups (eight control animals, eight animals with emphysema treated with a growth factor (LGF) and eight untreated animals with emphysema) and identified 1,700 DE mRNAs (90 over and 200 lower expressed). We hypothesized that certain dysregulated RNAs could exert their functions either individually or cooperatively, and some of them could be exploited as therapeutic targets. Zscan2 (zinc finger and SCAN domain-containing protein 2) and Bag6 (BCL2-associated athanogene 6) appear to have been identified as potential tissue repair players in an experimental model of COPD treated with growth factors based on lung gene expression.

**Zscan2** (also called ZFP29 or SCAND2P) is a transcription factor that plays a role in embryonic stem cell maintenance, telomere elongation in a small population of cells during embryonic development and genomic stability. This process helps maintain the integrity of the genome and allows for the preservation of stem cell populations [26]. Because of complex regulatory mechanisms, Zscan transcription factors may exhibit promoting or prohibitive effects on angiogenesis and cellular apoptosis. In addition to cell differentiation and proliferation, cell migration and invasion, the properties of stem cells and susceptibility to chemotherapy [27]. However, the role of Zscan2 in lung tissue repair and COPD is relatively less explored. This is the first study that might have discovered an association between Zscan2 expression and tissue repair processes in the lung affected by COPD. The exact mechanisms through which Zscan2 may contribute to tissue repair in COPD are not clear and require further investigation.

**Bag6** (also known as HLA (human leukocyte antigen)-B-associated transcript 3, BAT3 or Scythe) is a chaperone nucleocytoplasmic shuttling protein with several structural patterns. It is localized in the mitochondria and involved in controlling protein quality and regulating protein degradation pathways [28]. It interacts with various proteins, including misfolded or damaged proteins, to facilitate their proper folding, assembly, or degradation. Bag6 has been implicated in various cellular processes, such as apoptosis (from the formation of a caspase-3- cleaved Scythe C-terminal fragment), autophagy (mitophagy after mitochondrial depolarization), antigen presentation and T-cell response and NK cell activity in immune responses identified as a ligand for NKp30 involved in antitumor immunity of NK cells [29]. Scythe is critical for normal development and viability, probably through regulation of programmed cell death and cell proliferation [30]. The exact role of Bag6 in the lung is not yet fully understood, but there is evidence to suggest that it may play a role in regulating inflammation and immune responses in the lung. Bag6 appears to be a negative regulator in the innate immune system of non-small cell lung cancer patients [31, 32] and a risk factor for lung cancer [33, 34], even in early non-smoker lung adenocarcinoma patients [35]. Germline variants of genes involved in nuclear factor-kappa B (NF-κB) activation are associated with the risk of developing COPD and lung cancer [36].

Some COPD subtypes may be rare enough to be considered rare diseases (ORPHA: 101944). Even the presence of specific genetic mutations makes certain cases of COPD rare in the general population. Framing an experimental COPD model within the context of rare diseases requires an approach that combines both understanding the commonalities and investigating the unique factors that make certain cases of COPD rare. By integrating genetic, environmental and experimental considerations, it is possible to advance the diagnosis, treatment and management of these rare forms, thus improving the quality of life of affected patients.

## Conclusions

In summary, in our study, the lung genes Zscan2 and Bag6 seem to have been identified as potential tissue repair players in an experimental model of COPD treated with growth factor—LGF. Both genes appear to play a role in regulating inflammation and immune responses in the lung and targeting may be a potential therapeutic strategy for inflammation and injury-associated lung diseases. However, further studies are necessary to demonstrate this causal relationship.
